# A survey of methods for evaluating mini-publics

**DOI:** 10.1007/s41685-020-00183-3

**Published:** 2021-01-02

**Authors:** Takeuchi Ayano

**Affiliations:** Department of Environmental Science, Faculty of Science, Toho University, 2-2-1 Miyama, Funabashi, Chiba 274-1252 Japan

**Keywords:** Evaluation of mini-publics, Public participation in policymaking, Deliberative democracy

## Abstract

Public participation has become increasingly necessary to connect a wide range of knowledge and various values to agenda setting, decision-making and policymaking. In this context, deliberative democratic concepts, especially “mini-publics,” are gaining attention. Generally, mini-publics are conducted with randomly selected lay citizens who provide sufficient information to deliberate on issues and form final recommendations. Evaluations are conducted by practitioner researchers and independent researchers, but the results are not standardized. In this study, a systematic review of existing research regarding practices and outcomes of mini-publics was conducted. To analyze 29 papers, the evaluation methodologies were divided into 4 categories of a matrix between the evaluator and evaluated data. The evaluated cases mainly focused on the following two points: (1) how to maintain deliberation quality, and (2) the feasibility of mini-publics. To create a new path to the political decision-making process through mini-publics, it must be demonstrated that mini-publics can contribute to the decision-making process and good-quality deliberations are of concern to policy-makers and experts. Mini-publics are feasible if they can contribute to the political decision-making process and practitioners can evaluate and understand the advantages of mini-publics for each case. For future research, it is important to combine practical case studies and academic research, because few studies have been evaluated by independent researchers.

## Introduction

To consider solutions to complex and uncertain problems, such as environmental management, city planning, and health risk issues, the public participation approach has become increasingly necessary to connect diverse bodies of knowledge and a wide range of values to agenda setting, decision-making, and policymaking (Eden 1996; Few et al. 2007; Den Broeder et al. 2018). For instance, in the city planning field, Rittel and Webber (1973) distinguished between two types of problems, “tame problems” and “wicked problems.” Tame problems are clearly defined with known solutions whereas wicked problems are more difficult, with unknown solutions. The reason such solutions are unknown is because all possible solutions must consider citizens’ lives, values, and cultures. For example, climate policy and rules after the COVID-19 crisis have to consider a balance between the individual citizen’s life and the regulations that have to be followed by every member of society. These kinds of phenomena are often seen in areas, such as health communication and environmental issues (Zhang and Kim 2016; Mertens 2015).

Addressing these more complex and uncertain problems requires processes where various stakeholders, who are influenced by or may influence the decision, discuss the issues and create recommendations for policy formation (Rowe and Frewer 2000; Abelson et al. 2003; Fung 2006). It has also been pointed out that when the views and values of the citizens are not fully integrated into the deliberative process, it can lead to political distrust (Pellizoni 2011).

One approach to integrate citizens’ views and voices into the political decision-making process is deliberative democracy. According to Risse (2004), “Deliberation is based on arguing and persuasion as non-hierarchical means of steering to achieve a reasoned consensus rather than a bargaining compromise.” Deliberation refers either to a particular sort of discussion—one that involves the careful and serious weighing of reasons for and against some proposition—or to an interior process by which an individual weighs reasons for and against courses of action (Fearon 1998). Because wicked problems are characterized by the lack of a single known solution or set of solutions, solving them requires broader participation by all involved, including citizens. As a means of securing citizens’ participation in solving wicked problems, mini-publics are gathering attention.

Mini-publics are an effort by which randomly selected citizens (hereinafter “participants”) are provided with information and they form opinions on a subject by discussing it over several days within a particular framework. The development of mini-publics’ methodologies started in the 1970s, and throughout the years, a variety of them have emerged. The following major methodologies comprise the focus of this paper.

### Consensus conference

Developed by the Danish Board of Technology in 1985 as an attempt to involve the public in technology policy, this methodology includes a citizen panel. It is seen as a form of technology assessment and usually includes dozens of citizens, who undergo four processes: (1) weekend meetings, usually lasting 2 to 3 days, for understanding the context of the theme; (2) deliberations, usually over 4 days, led by facilitators; (3) interaction with citizen panels and experts; and (4) policy recommendations (Kleinman et al. 2009).

### Deliberative polling

Developed by Robert C. Luskin and James S. Fishkin in the early 1990s, this methodology incorporates deliberation into the conventional opinion poll (Luskin et al. 2002) and usually includes hundreds of participants. Participants receive information on a proposal which they are encouraged to discuss within groups and vote on over 2 to 3 days. Like traditional opinion polls, the results of deliberation-type opinion polls are an aggregation of individual opinions (not a consensus), except that in the case of deliberative polling, individual opinions are formed after group deliberation.

### Citizens’ jury/community jury

The citizens’ jury was developed by Crosby (1995), who promoted and organized this type of mini-publics at the US state government level in agricultural, water, and welfare policies. Community jury is a community-adopted version of citizens’ jury. Juries made up of 12–24 randomly selected citizens receive information, learn about evidence, cross-examine experts and witness, and discuss the issues at hand over 2 to 3 days (Crosby 1995).

### Planning cell

The planning cell was developed by Peter C. Dienel in the 1970s (Dienel 2002). Deliberations are held among cells of approximately 25 randomly selected citizens. Each cell is then divided into small groups of four–six people who receive information, discuss the issue, and summarize the participants’ opinions as citizen reports. Since accountability requirements are built into the process, sponsors must agree to consider the decisions made by the planning cell.

These forms of mini-publics are expected to provide a common understanding by lay citizens through deliberation. The legitimacy of mini-publics is often questioned because they afford unelected citizens the privileged status of shaping public policy (Lafont 2015). Nonetheless, their popularity is increasing year by year, and in each case, evaluations of the proceedings are made by practitioners from the implementing organizations, which include universities and research institutes. However, while practitioners mostly focus on the implementation, their results are not analyzed by independent researchers. The current author’s motivation is to further advance mini-publics development by analyzing the relationship between good local practice and high-quality research evaluation. There are various types of mini-publics as outlined above, and they are experimentally implemented at the local and national levels depending on budget, human resources, and purpose (Mannarini and Fedi 2018). For this reason, the results obtained are different in each case, and various effects are intertwined in a complicated manner, so a comprehensive evaluation has not yet been made (Abelson et al. 2003). The evaluative process is not based on judgments of good or bad but is driven by the idea that learning is an action to gain something (Petts 2001), and thus, many successful cases have accumulated. Such analyses are expected to lead to the construction of a better system.

To start this discussion, the author undertook this literature review to present a high-level picture of recent mini-publics evaluation research conducted by both independent researchers and practitioner researchers, aiming to capture a unified view of the evaluation of mini-publics in practice. By not limiting the focus to a specific theme, such as the environment or health, it is possible to investigate the common features across a wide variety of themes. Although each theme is different, identifying common phenomena will support the institutionalization of mini-publics as a standardized public participation approach or a minimum requirement of a public participation approach.

## Methodology

### Search strategy

This survey of mini-publics was conducted in December 2019. The author searched the Web of Science Core Collection, the BIOSIS Citation Index, the Current Contents Connect, the Data Citation Index, the Derwent Innovations Index, and the MEDLINE database for documents that included the terms “mini-publics,” “consensus conferences,”^1^ “deliberative polling,” “citizens’ jury” “community jury,” and “planning cell” with the combination of the terms “evaluation” and “assessment” in the title, abstract, and keywords. The scope was limited to empirical case studies written in English and published in peer-reviewed journals between 2004 and 2019. The initial search found 123 articles. Screening the titles and abstracts and removing duplicate and non-English language papers left 29 papers.^2^ In screening, the author confirmed that each of the 29 papers was an empirical case study of mini-publics. In some of the studied cases, for certain themes, the population is not based on national or municipality level units, for example, groups based on age; all those with randomly sampled participants from a population will be considered to be conducting mini-publics.

### Diversity of themes

The 29 literature reviews were classified as follows: healthcare (which treats issues related to lifestyle and also health technologies; eight studies), environment and social (eight studies), medical care (includes the screening of diseases and ethical issues; nine studies), and food (four studies).

#### Healthcare

Mini-publics prioritize and allocate resources to health technologies and health and social care in communities (Gooberman-Hill et al. 2008; Menon and Stafinski 2008; Stafinski et al. 2012; Bonbard et al. 2011) and to required health services (Kashefi and Mort 2004; Bond et al. 2016). As a result of examination, deliberation-style exercises in democracy were employed. Some studies were focused on specific health themes, including the health impacts of youth anti-social behavior in communities (Haigh and Scott-Samuel 2008). Other deliberations included developing a regional framework for the health impact of food-related multinational companies, such as McDonald's (Anaf et al. 2018).

#### Environment and society

Regarding the theme of environment/society, the analyzed mini-publics were primarily in the field of urban planning. In recent years, problems that have been previously treated as tame by experts have been increasingly regarded as wicked problems; mini-publics on road maintenance, regional planning, and gasification plant installations were carried out (Franceschini and Marletto 2015; Lundström et al. 2016; Mannarini et al. 2018). Deliberations on the use of water resources allocation with biological and sociological interests were also undertaken (Straton et al. 2011). The role of citizens in the development of science and technology and the risks and policies therein have also been studied; other topics regarding new technologies and the risks of their implementation include animal welfare (Miele et al. 2011) and nanotechnology (Kleinman et al. 2009). Furthermore, other political issues, including financial delegation, immigration, the state of government (Mannarini et al. 2018; Felicetti et al. 2015), EU integration, and climate change (Smets and Isernia 2014), have been evaluated.

#### Medical care

Under the medical care theme, the author encountered and analyzed a host of controversial topics including the availability of health screening and age restrictions, cystic fibrosis screening (Mosconi et al. 2018), controversial prostate-specific antigen screening (PSA; Rychetnik et al. 2014), controversial designer DNA testing for diseases in unborn babies (Iredale and Longley 2007), prioritization of immunization programs (Parrella et al. 2016), and mammography examinations of women between 70–74 years old (Degeling et al. 2018). For example, regarding PSA screenings, while there is a possibility of increasing the survival rate, there is also concern that unnecessary treatment of prostate cancer may increase the burden on the body (Rychetnik et al. 2014). Another study on the prioritization of adolescent immunization programs was based on a sample from the youth population; this is notable because the opinions of young people who are actually vaccinated are often overlooked (Parrella et al. 2016). These approaches target the subject whose opinion is difficult to reflect.

In addition, many themes that deal with ethical issues were taken up and carried out with the aim of reflecting citizens' value judgments, including Xenografts, the first internationally discussed topic (Jones and Einsiedel 2011; Hansen and Allansdottir 2011), and euthanasia or assisted dying (EAD), a controversial issue in medical ethics and healthcare policy (Walker et al. 2019). The value of a biobanking policy to store human biological specimens and data for research purposes was also deliberated (Walmsley 2011).

#### Food

Regarding food, the consensus meeting discussed the most frequently considered topics regarding genetically modified organisms (GMOs; Tavella 2016; Dryzek et al. 2009) and the most effective form of advertising dangers of the retail food industry to avoid its negative effects on the public health (Timotijevic and Raats 2007; Henderson 2013).

### How to review the evaluation research

For this research, the author proposes the following framework with a matrix of evaluator category and evaluated data to classify the reviewed studies.

The evaluators are divided into two categories: (1) Practitioner researchers who conduct mini-publics practices for entities including private organizations, universities, and governments; and (2) independent researchers who do not participate in the design process of mini-publics. Evaluation by practitioner researchers can be very detailed and structured, because it is possible to develop the evaluation scheme before the mini-publics is conducted and to develop tailored concepts for evaluation. However, the possible existence of evaluator bias among practitioner researchers has been pointed out (Azzam 2011). Independent evaluation can prevent bias and is also indispensable for avoiding manipulation by interested parties and for clarifying that the original value and purpose of the exercise was maintained. In reality, it is not possible to clearly distinguish practitioners and independent evaluators because in some cases, independent evaluators are involved in the process of mini-publics conducted for entities. But in those cases, it was possible for the evaluators to mention the former setting of evaluation, so the author placed such evaluators in the practitioner category.

Evaluated data are generally divided into two approaches, qualitative and quantitative research. Recently, the integration of the two has been formalized as a multi-strategy research expected to deepen the researcher’s understanding of the research question (Bryman 2006). In the present research summarizing the evaluation concept of public participation, the author proposes a framework for reviewing the papers. Qualitative research is empirical research where the data are not in the form of numbers (Punch 1998); two existing relevant approaches to qualitative research exist are discussed in the present paper.

#### Participation process evaluation

There are several evaluation frameworks and indicators that measure the effectiveness of public participation. Grounded theory, a well-known social science methodology, aims to develop a theoretical analysis of the data that both fits the data and has relevance to the area of study (Charmaz 1996); it is widely used in the evaluation of the public participation process. Because individual methods perform differently in different political contexts and according to a range of factors (Abelson et al. 2003), it is necessary to compare many cases to create effective standards and rules. Webler (1995) developed the widely used evaluation concept of “Fairness and Competence,” and using grounded theory, he compared the meaning and relevance of categories and concepts that emerge out of a systematic coding process (Webler and Tuler 2000). It should be clarified concretely which methods of public participation are most effective using social science indicators built especially for these evaluations (Rowe and Frewer 2000; Gooberman-Hill et al. 2008; Abelson et al. 2003). Representation, respect, justification for rebuttal, and rationality for deliberation should be consider when evaluating public participation in deliberation processes.

#### Deliberation contents evaluation

In addition to the frameworks and indicator methodologies, “discourse analysis” is used as an evaluation method. It aims to understand how people use language to create and enact identities and activities (Starks and Trinidad 2007). Protocol analysis, a well-known method based on verbal reports and qualitative data analysis software, such as NVivo, is used to measure the quality of deliberations. Coding methodology representing a discourse quality index (Steenbergen et al. 2003), which includes coding for participation, level of justification, content of justification, respect for the group, respect for requests, respect for rebuttal, and constructive politics, is also applied.

#### Quantitative Data Evaluation

Quantitative research gathers data in a numerical form which can be categorized, or ranked in order, or measured in units of measurement; the methodologies of quantitative research are summarized experiments, surveys, and predetermined instruments (Bahari 2010). The process is generally expected to produce a change in participants’ attitudes including values, views on issues, causal assumptions, ideologies, and specific policy attitudes; such changes can lead to people being more inclined to seek information and discuss issues (Fishkin 2013; McDevitt et al. 2006).

## Results

The 29 articles are divided into four categories by combination of research type and evaluation approach (Tables 1 and 2). Of the 29 mini-publics studied, 21 cases are introduced with the citizens’ jury/community jury method, and six cases with the consensus conference and technology assessment method. There is only one deliberative polling case, and there are no cases evaluating the planning cell method. On the practitioner base, practical evaluation reports were published by evaluators in each category; however, research evaluation was not located for deliberative polling or the planning cell.

**Table 1:** Categorization of reviewed papers

Evaluator	Evaluated data		
	Qualitative data		Quantitative data
	Participation process	Deliberation contents	
Practitioner	Evaluation of feedback from participants	Evaluation of deliberation contents	Questionnaire Survey
Independent	Feedback from participants and organizer report	–	–

**Table 2:** Reviewed papers

	Themes	Method	Evaluation	Evaluation Focus	Results/points
Kashefi and Mort (2004)	HC	CJ	Theoretical framework	Feasibility of CJ	Local organization, listening skills for politics, and clear vision for integration into the political decision-making system
Gooberman-Hill et al. (2008)	HC	CJ	Theoretical framework	Deliberative condition and participants	Interest, conflict, and harmony of deliberation
Kleinman et al. (2009)	ES	CC	Theoretical framework	Motivation of citizens	Information, enough time for deliberations
Haigh and Scott-Samuel (2008)	HC	CJ	Theoretical framework	Feasibility of CJ	New input can be obtained
Bond et al. (2016)	HC	CJ	Theoretical framework	Feasibility of CJ	New focus (from indigenous people), new input can be obtained
Iredale and Longley (2007)	MC	CJ	Manuscript	Is it possible to use CJ as social research?	Reliable platform
Franceshini and Marletto (2015)	ES	CJ	Manuscript	How CJ works, combined model	Early introduction of CJ
Lundström et al. (2016)	ES	CJ	Multidimensional model	Deliberation will change the storyline	Topic, facilitator, clear vision for integration to the political decision-making system
Jones and Einsiedel (2011)	MC	CJ	Theoretical framework	Institutionalization	Transparency and Accountability
Tavella (2016)	Food	TA	CHS approach	Feasibility of TA	Variety of interests and values concerning of stakeholders are integrated
Dryzek et al. (2009)	Food	CC	Document research, Interview	Political context and mini-publics	Difficult to legitimate the deliberation with precautionary worldviews
Hansen and Allansdottir (2011)	MC	TA	QCA	Factors relate to policy formation	Strong factors are listed
Felicetti et al. (2015)	Food	Others	Document Research	Political context and mini-publics	Separation between public discourse and decision-making process
Mannarini and Fedi (2018)	ES	CJ	Manuscript, Questioner, interview	Quality of deliberation	No innovative deliberation, expert opinion, minority opinion
Walmsley (2011)	MC	CJ + CC	Action Research	Disagreement plays important role in dynamics	Improved the quality of deliberation by disagreement
Anaf et al. (2018)	HC	CJ	Action research	If CJ can develop citizen-informed policy recommendations	Representative, neutrality of expert
Degeling et al. (2018)	MC	CJ	Action research	Elicit informed view	Representative, expert
Menon and Stafinski (2008)	HC	TA	Manuscript	Feasibility of CJ	Participants’ opinion is different from experts’
Parrella et al. (2016)	MC	CJ	Action research	assess young people’s views	Independent facilitator, lack of best practice summary
Miele et al. (2011)	ES	CJ	Action Research	Open technical and scientific issue to the public	The results are partly reflected in the final document
Straton et al. (2011)	ES	CJ	Action Research	Open technical and scientific issue to the public	Deepen citizens’ understanding
Rychetnik et al. (2014)	MC	CJ	Questionnaire survey Content analysis	What are public values and concerns?	Information provision Enough information, deliberation change opinions
Mosconi et al. (2018)	MC	CJ	Questionnaire survey Content analysis	Difference between CJ and online survey	Enough information
Bombard et al. (2011)	HC	TA	Questionnaire survey	Ethical and social value input	Values were prioritized
Timotijevic and Raats (2007)	Food	CJ	Questionnaire survey	Comparing workshop and citizens jury	Group deliberation is important for satisfaction, theme should be clear
Henderson et al. (2013)	Food	CJ	Questionnaire survey	Feasibility of CJ	Enough time for deliberation, information provision = satisfaction, facilitator
Stafinski et al. (2012)	HC	CJ	Questionnaire survey	Short and long impact	Information sharing continued for 6 weeks
Walker et al. (2019)	MC	CJ	Questionnaire survey	Opinion change	Sufficient time for deliberation
Smets and Isternia (2014)	ES	DP	Questionnaire survey	Attitude change	Predisposition has influence

*HC* Health Care, *ES* Environment and Society, *MC* Medical Care, *CC* Consensus Conference, *DP* Deliberative Polling, *CJ* Citizens’ Jury or Community Jury

### Evaluation of feedback from participants

This category features seven cases evaluated by practitioner researchers which focus on the participation process with feedback from participants to discuss the overall design of mini-publics. Some were conducted as part of social surveys and others were conducted in collaboration with the community. Relatively many cases of mini-publics were organized in small communities.

Kashefi and Mort (2004) evaluated written and face-to-face feedback from participants and stakeholders in a framework of deliberation, integration, sustainability, and accountability and integration with existing local organizations, building the listening skills of local parliamentarians, and a clear vision of how the post-deliberation process works to avoid a bureaucratic pre-occupation problem.

Gooberman-Hill et al. (2008) evaluated participant feedback forms and explained how the deliberative conditions affect participants and noted that interest levels may be related to jury members’ interest and belief in their ability to shape future discussion; the article also stated that conflict and harmony were important parts of the deliberation process and suggested helping participants exchange experiences. A study that evaluated the data of semi-structured interviews to clarify participants' motivations for participation was found to depend on the information provided during recruitment. For example, if the public is informed that consensus meetings are a novel method of citizen engagement, people who are interested in citizen participation will gather. If the public is informed that they could learn about technical issues, people who are interested in technical topics will gather (Kleinman et al. 2009). Although the initial introductions were varied, similar conclusions were reached after information and deliberation (Kleinman et al. 2009).

In other notable findings regarding participants’ feedback, Haigh and Scott-Samuel (2008) described how participants revealed relevant insights about the topic which had not been focused on in the past, and Bond et al. (2016) introduced a community jury in Australia to involve indigenous people in health research and noted that it gave them the advantage of contributing to the construction of health services in their community. Case studies of how citizens’ juries can be used in social research revealed data on the phenomena of interest that are difficult for decision-makers to ignore. Researchers have suggested that providing a reliable platform to discuss and consider issues creates opportunities for the general public to enter the policy process (Iredake et al. 2007). In Franceshini et al. (2015), the citizens’ jury was used in a combined model with a stakeholder meeting; the combined model was effective as a method of avoiding the manipulation of proceedings by stakeholders, but the jury was introduced late in the deliberation. It was pointed out that this led to the participants’ frustration.

Studies that evaluate participants’ feedback show that mini-publics can help deconstruct the existing decision-making scheme and point out the necessary conditions for this to happen. These conditions range from the level of integration with the political decision-making system and locally embedded organizations, deliberative conditions, such as a well-skilled facilitator, neutrality of information, and general timing to the length of the mini-public.

### Feedback from participants and organizer report

Qualitative evaluation was used in six cases involving participatory process reports. The purpose of these cases was to examine the integration between the mini-publics practice and the political decision-making process from a third-party perspective.

A study of participant interviews, which developed a “multidimensional evaluation model” with the factors of content dependence, stakeholder role, multi-tiered data, and development of criteria, demonstrates that the accuracy of and interest in deliberation topics and well-skilled facilitators are important for an effective process (Lundström et al. 2016). Lundström et al. (2016) contend that a clear vision for integration into the political decision-making system should ensure that citizens play active roles and do not remain only audience members; it is challenging to handle people who are inexperienced at participating in a deliberative process and justifying their opinions. Jones and Einsiedel (2011), in a study that analyzed semi-structured interviews with policy-makers and examined the institutionalization of the mini-publics method, noted that rational deliberations among various stakeholders took time but produced valuable results, including a new mechanism for ensuring transparency and accountability in decision-making process. Critical Systems Heuristics (CSH) is a tool consisting of 12 questions that clarifies the daily decisions we rely on at the conscious and subconscious level to understand situations and design systems to improve them; a study using CSH demonstrated that mini-publics allowed the assessment of GMOs to include a variety of interests and values of concern to stakeholders (Tavella 2016).

On the other hand, studies comparing country-by-country reports and interviews on initiatives under the same theme have shown that mini-publics can lead to a precautionary world view in contrast to the governmental “Promethean outlook.” This can result in the “boundless faith in the capacity of humans to manipulate complex systems for their own advantage,” and results revealed concerns that the reasons behind the government’s support for mini-publics were unclear and that government-supported mini-publics could be manipulated (Dryzek et al. 2009).

Hansen and Allansdottir (2011) used relevant materials to clarify the conditions that lead to restrictive policies regarding Xenotransplantation and those that lead to innovative policies. Comparing 11 Western European countries; a qualitative comparative analysis (QCA) was employed. The QCA aims to identify the necessary and sufficient conditions for the results to occur and to eradicate the least important factors, thereby identifying the composition of factors that can best explain the results of interest. For example, in Hansen and Allansdottir’s (2011) research, “relevant Xenotransplantation policy,” “place of public participation,” “political participation,” “public concerns,” “business,” and “scandals” were listed as requirements for inclusion.

Felicetti (2015) conducted qualitative and interpretive analyses (Stevenson and Dryzek 2012) which was used in Dryzek’s (2009) conceptualization of deliberation capacity building (inclusiveness, certainty, necessity) to compare cases introduced in different political contexts with different designs; the common feature of the deliberation campus is that many participants attempt to join in discussing a wide range of topics. This produces results without refinement and justification and is therefore separated from general political discourse.

In contrast, independent researchers tend to take a more holistic view toward the evaluation of mini-publics, including the impact on actual policymaking. From the perspective of political integration, mini-publics might be a tool to influence participants.

### Evaluation of deliberation contents

Qualitative analysis was used in eight cases to evaluate the deliberation contents. The main purpose of these studies was to explore the value of introducing civic opinion by comparing it with conventional policies and expert opinions and analyzing the effects of disagreement between participants. Two studies visualized the result using quantitative indicators. NVivo software was used for content analysis.

Mannarini et al. (2018) investigated the quality of deliberation using the number, length, and contents of remarks during deliberation based on the concepts of dominance, cooperation, and openness of recognition and observed that comments introducing innovative knowledge and deliberation were not observed. Expert opinion was cited as a factor in changing participants’ opinions, and the quantitative balance of various opinions should be maintained for better deliberation quality. Walmsley (2011) adopted a qualitative analysis approach that separates utterances by speaker. The study considered the role that disagreement among participants can play in the dynamics of the deliberation, and observed participants adapting their own opinions after listening to those of others. It was noted that this process contributed to improving the quality of the deliberation.

The following four cases investigated whether it is possible to develop citizen-informed policy recommendations using mini-publics. Anaf et al.’s (2018) case study on the health impact of fast food suggested that strict regulations be placed on advertising and consumer information standards related to it. In Dageling et al. (2018), a community jury convened to discuss the availability of mammography screenings for older women and demonstrated the value in providing information to these women, allowing them the chance to make their own decisions and taking conservative measures until evidence is provided. Menon and Stafinski (2008) investigated the feasibility of the citizens’ jury by objectively weighting the basis for prioritization using the minutes of deliberations; the study revealed a significant difference between participants and experts regarding perceived future costs of health technologies. Parrella et al.’s (2016) study regarding prioritizing government funding for youth immunization programs has led to important social implementation recommendations for policy-makers, including the recommendation that immunizations be provided along with information and education. These cases mentioned that representatives, neutrality of experts, and independent facilitators are required.

In the final two cases in this category, Miele et al. (2011) and Straton et al. (2011) covered themes relating to animal welfare improvement strategies and water resource allocation strategies, respectively; these studies were implemented in a combined model with stakeholder meetings. Based on the opinions expressed during the stakeholder meetings, standards and alternative scenarios were developed, the outcomes of which were discussed by citizen juries, and recommendations for social implementation were drawn.

The evaluation of deliberation contents focuses on citizens’ participation: most studies evaluated mini-publics as positive, because they highlight different aspects of existing policy. The same studies pointed out that citizen deliberation cannot be innovative, unless there is disagreement between participants and an independent facilitator is able to support the creation of appropriate deliberation conditions.

### Questionnaire survey

In eight cases, researchers evaluated the pre- and post-deliberation questionnaire surveys quantitatively and the content of the deliberation qualitatively to complement. Questionnaire surveys in these cases were used to obtain participants’ demographic data (such as age, gender, ethnicity, religion, education, employment) and their feedback on motivation and process. Questionnaires also investigated whether participants’ opinions changed after the deliberation. The purpose of analyzing the deliberation content was to identify the core values derived from the process and explore the grounds for participants’ opinions as revealed by the questionnaires. All deliberations in the eight studies were recorded and transcribed.

Participants and control groups were compared in parallel cases to investigate opinion change through deliberation. In Rychtenik et al.’s (2014) study on the theme of PSA screenings, the same questionnaire was administered to the jury and the control group; both participated in the information session, and only the jury deliberated. The result shows that the jury believed that they had more knowledge about the advantages and disadvantages of PSA screening than the control group; the jury recommended alternative government programs to support general practitioners in providing enough information to the public to allow them to consider positive and negative impacts and make an informed decision. Mosconi et al.’s (2018) case regarding cystic fibrosis carriers screening demonstrated that even using online surveys, it is possible to deliberate broad and interesting insight into public perceptions and opinions if the jury is provided enough information.

In cases where attention was paid to changes in participants’ opinions, questionnaires were administered three times before the start of the session, once after providing information, and once after deliberation, or twice before and after the start of the session, and the differences between each questionnaire were analyzed. Bombard et al.’s (2011) case on health technology assessments found no significant change from pre- to post-deliberation. In Timotijevic and Raats’ (2007) study regarding food-policy development, the average value (five levels) was calculated and a t-test performed, but no significant change was observed between pre and post-questionnaires. However, some interesting results were yielded upon analysis of the deliberation content. For example, participants tended to stick to their opinions when the theme of the deliberation was ambiguous (Timotijevic and Raats 2007). By contrast, in Henderson et al.'s (2013) study of food advertisements at a children’s event—highlighting the need for deliberate sampling of the minority because of the limited diversity of representativeness from attribute analysis—post-deliberation questionnaires revealed that 82% of the participants appreciated the increased knowledge. Deliberation tended to be affected by the content of the information provided, but the effect was reduced if enough time was taken for deliberation. However, some participants continued deliberations after agreement and dominated the conversation, suggesting that the presence of a facilitator who could check and manage the timing was essential. In the case of Stafinski et al. (2012), the rating question uses the Wilcoxon paired signed rank test, and the preference question examines the probability of a participant changing their opinion and sets the significance of that percentage to 0.20. The analysis was performed with the type I error probability set to 0.05. As a result, it was observed that opinion change occurred due to the provision of information, and the change continued for six weeks. In Walker et al.'s (2019) case, opinions on EAD were polarized after deliberation. A summary of the reasons behind the polarization suggests that without enough time, it is not possible to propose a compromise on a theme that involves a complex range of personal value judgments, such as in the EAD deliberation, which was shown to be useful as a means of promoting understanding of the theme and clarifying the background of disagreement. Smets and Isernia (2014) used heuristic models to determine awareness (defined as the amount of attention we pay to politics and how much we understand what we know) and predisposition (defined as the acceptance or rejection of the political communications that people receive). Based on the hypothesis that variables with different knowledge play a major role in opinion change, a multiple regression analysis was performed with three variables, and the results were compared with the control group. Smets and Isernia (2014) discussed EU integration, climate change, and immigration, and noted that predisposition had the most influence on opinion change in all themes, and that information provision and deliberation did not have a significant effect. However, some themes, such as immigration reinforce previous ideas, while others, such as European integration and climate change, are more technical and sensitive to dissonant information after deliberation.

Multiple questionnaire surveys have investigated which factors influence participation and deliberation itself. These studies show that the participants recognized that their knowledge level of the deliberated theme had increased after deliberation. This enables them to create a new proposal different from the existing decision-making process. Sufficient deliberation time should be secured to avoid polarization of opinions. The theme setting is critical to ensure participants are able to deliberate without any bias. For example, themes containing participant bias, such as immigration, are mostly affected by the experiences and predispositions of its participants.

## Discussion and conclusion

In this study, the author reviewed 29 cases with the aim of presenting a high-level picture of recent mini-publics evaluations by both practitioner researchers and independent researchers, thereby obtaining a unified view of the evaluation of mini-publics in practice. The vast majority of case studies evaluated by practitioner researchers used the citizens’ jury. Citizens’ jury is a tried and tested methodology and has also been proposed for use as a social survey (Iredale and Longley 2007). This might be because the cost of a citizens’ jury is moderate compared to other methodologies. Mini-publics are a tool to approach issues that have no clear answer and where new insights by citizens can create a new dynamism in a democratic situation. However, sensitive issues, such as minority treatment, immigrants, or indigenous people, might pose difficulties in creating a new dynamism due to the risk of influence of participants’ bias.

### Qualitative studies

Research developed mainly by practitioner researchers tended to focus on the positive aspect of mini-publics and clarified the conditions under which ideal mini-publics can thrive. To maintain a better quality of deliberation, the following observed common features are important: neutrality and sufficiency of information and expert input; the existence and balance of different opinions including of minorities and independent and skilled facilitators; and the capacity development of participants. Research evaluating deliberation contents was critical of the fact that innovative deliberations had not been observed.

Independent researchers tended to focus on the development path between the practice of mini-publics and political decision-making processes, as well as a lack of concern about the capacity of participants to participate in deliberations separate from prevailing political discourse. To develop a pathway to the political decision-making process, it is important that policy-makers and experts consider the quality of deliberations. Independent researchers also compared various cases to generalize the features of the citizens’ jury and understand the political and cultural contexts of the location of mini-publics. The studies implemented by researchers were mainly technology assessments and included a consensus conference.

### Quantitative data research

Quantitative research was mainly questionnaire surveys and conducted by practitioner researchers, because it is related to the design of mini-publics practice. For example, in cases where opinions had changed, such changes were thought to result from the predispositions and beliefs of participants, while some studies showed that the provision of information by experts also had a significant effect. Factors that affect opinions vary from case to case, so they should be demonstrated by quantifying qualitative data rather than using questionnaires and exploring common terms while organizing initial settings and results in various cases, as necessary. This means that participants’ bias remains, so researchers would have to consider how to choose participants, highlighting the importance of technical neutrality in the development of the process.

To promote better mini-publics practice in response to wicked questions, it is important to systemize the elements of good practice demonstrated in each case study from a pragmatic perspective. However, such evaluation research depends on the political and cultural context, and it is challenging to evaluate these differences as practitioner researchers. The limited number of studies where practitioners (not affiliated to a research institute) and researchers collaborated is attributed to the complicated nature of such collaboration: researchers tend to focus on the meaning of mini-publics itself, while practitioners consider the relative success of each mini-public. Empirical investigation is required to understand the deliberative practice functions of the whole process (Elstab et al. 2016).

Future research should examine findings from both practical case reports and independent researchers, along with insights gained from networks, such as Democracy R&D or Participedia (Smith et al. 2015). Such an approach will promote cooperation between practitioners and independent researchers, ultimately enabling the practice of mini-publics to improve the status of global democracy. Such research can also contribute to the further development of best practices in the context of mini-publics. This study does not cover cases that do not include the terms evaluation and assessment, and it is necessary to conduct a broader review as mini-publics are often evaluated at the practical level.

## Footnotes

1 Because a search for cases of medical consensus meetings returned voluminous results, the term “citizen” was added to narrow the search.

2 The 26 papers which I could not obtain are excluded.
